# Deterioration of initially accepted radiological alignment of conservatively treated AO type-C distal radius fractures: mid-term outcome

**DOI:** 10.1007/s00590-020-02659-6

**Published:** 2020-03-26

**Authors:** Leena Raudasoja, Heidi Vastamäki, Samuli Aspinen

**Affiliations:** 1 Department of Musculoskeletal and Plastic Surgery, Helsinki University Hospital and University of Helsinki, PL266, 00029 Helsinki, Finland; 2 Sports Trauma Research Unit, Hospital Mehiläinen Neo, Turku, Finland

**Keywords:** AO type-C, Distal radius fracture, Conservative treatment, Mid-term outcome, Patient-rated outcome

## Abstract

**Background and aims:**

It still remains controversial how often the once-accepted radiological alignment of an AO type-C distal radius fracture deteriorates after conservative treatment, and to what extent this deterioration is perhaps associated with patient-rated outcome measures (PROms). Thus, we aimed to evaluate this radiological deterioration and its association with mid-term functional follow-up.

**Patients and methods:**

We retrospectively reviewed 66 patients (mean age at fracture 53 years, SD 14.1, range 18–73, female 65%) with 68 C-type distal radius fractures at a mean of 6.7 years (SD 0.5 years, range 5.8–7.7 years) after primary closed reduction and cast immobilization. Radiographs of the wrists were taken and analysed for any radial shortening, dorsal tilt or step-off at the joint surface. Range of motion and grip strength were measured. In addition to the radiological result, primary outcome measures included Quick Disabilities of the Arm, Shoulder and Hand (QDash) and Patient-Rated Wrist Evaluation (PRWE).

**Results:**

At mid-term follow-up, an acceptable anatomical radiological result was seen in only 22 wrists (32%). Deterioration of the once-achieved and accepted primary alignment was seen in a majority of cases (68%). Radial shortening of ≥ 2 mm was found in 34 wrists (51%, mean 4 mm, range 2–8 mm), with no association with QDash (12.8 vs. 5.5, *p* = 0.22) or PRWE (9.1 vs. 5.7, *p* = 0.40). Only four patients (6%) showed step-off at the joint surface (mean 1.1 mm, range 0.5–2 mm). Twenty-two wrists (32%) showed dorsal tilt of ≥ 10° (five with volar tilt of 15°–25°), with no effect on QDash or PRWE (14.7 vs. 6.5, *p* = 0.241 and 10.1 vs. 5.8, *p* = 0.226). Altogether, patients with dorsal tilt, step-off or shortening did not show significantly worse QDash (10.3 vs. 5.7, *p* = 0.213) or PRWE (8.1 vs. 5.1, *p* = 0.126) versus those with none. Twenty-nine (43%) of the patients had deficits in range of motion (ROM), either in extension (39%), flexion (43%), supination (16%) or pronation (4%), or combinations of these. Worse extension was associated with worse QDash (15.9 vs. 5.0, *p* = 0.037), flexion deficit with worse PRWE (11.5 vs. 4.4, *p* = 0.005) and supination deficit with both QDash (21.7 vs. 6.8, *p* = 0.025) and PRWE (18.9 vs. 5.2, *p* = 0.007).

**Conclusions:**

The initially accepted radiological alignment of AO type-C radius fractures deteriorated in a majority of cases during conservative treatment. However, this deterioration was fairly mild and showed no significant association with functional outcome. Restricted ROM showed some association with PROms.

**Level of evidence:**

IV.

## Introduction

Distal radius fractures represent one of the most common types of fracture, accounting for 18% of all fractures [1]. Successful closed reduction restoring the shape of the distal radial epiphysis constitutes the premier aim when selecting between conservative and operative treatment [1, 2]. With ageing of the population, the incidence of distal radius fractures is increasing constantly. Although the majority of distal radius fractures are treated successfully with conservative means, there is a trend towards surgical treatment [3].

Many reports show that better anatomical reduction (with operation) leads to better functional outcome [4, 5]. In particular, AO type-C fractures, which are considered to be at least relatively unstable [6], are commonly treated operatively. On the other hand, especially among elderly people, it has been suggested that radiological or anatomical outcomes do not correlate with clinical outcome or patient satisfaction. In addition, patients are predisposed to complications of surgery [7–9], which are not rare. The influence of regaining normal anatomy is not clear when it comes to clinical outcome [10, 11].

After closed reduction, re-displacement during conservative treatment is frequently seen as the reason for unfavourable functional outcome [2, 12]. However, some authors have pointed out the importance of near-anatomical reduction in a distal radius fracture, while others have stated the opposite, and elderly patients in particular may show satisfactory functional results despite imperfect anatomical healing [7, 13–15].

Finnish national Current Care Guidelines [11] direct treatment recommendations, especially in patients of working age. However, the increasing number of active elderly people in particular has led to the need to customize these recommendations to allow the best possible outcome of fracture treatment at each activity level and in each age group. It is easy to recommend surgical treatment for patients with severely displaced or notably re-displaced distal radius fractures, but there is a subgroup of patients that does not necessarily benefit from surgical intervention. AO classification type-C fractures show a tendency towards malalignment and instability during cast immobilization [16, 17]. Thus, we wished to investigate how intra-articular AO classification type-C distal radius fractures which were treated conservatively in our institute preserved their radiological alignment, and whether or not the possible deterioration would be associated with mid-term functional outcome.

## Patients and methods

The study design was a retrospective, mid-term follow-up case series study of 66 consecutive patients (68 wrists) with conservatively treated AO type-C distal radius fractures. All patients were primarily treated in 2010–2012. The research protocol was approved by Helsinki University Hospital Ethics Committee (DNRO HUS/330/13/02/2012) and conducted in accordance with the Declaration of Helsinki. After written information, a consent document was obtained from all participants.

All patients enrolled were treated in a University Hospital with a catchment area of 1.7 million inhabitants. This level I trauma centre is a referral hospital in which 500–600 distal radius fractures are treated annually. During the study period (2010–2012), a total of 982 radius fractures were treated operatively, of which 50–55% were AO type-C distal radius fractures.

Between 2010 and 2012, 138 patients with AO type-C distal radius fractures were treated by means of closed reduction and cast immobilization in our trauma unit. These patients were invited to take part in a follow-up study in 2017–2018. By then, seven of these patients had died, and 59 patients were unable to come (health issues, *n* = 6, had moved faraway or abroad, *n* = 17, cancelled, *n* = 11, declined to participate, *n* = 16, unknown reason, *n* 9. Six other patients were excluded (sought operative treatment in a private clinic, *n* = 4, pregnant, *n* = 2). Altogether, 66 patients (68 wrists, 48%) came to a follow-up visit 6.7 years (SD 0.5 years, range 5.8–7.7 years) after the initial treatment. Of these, 41 (62%) patients had low-energy fractures (falling from standing height or less than one metre) and 25 (38%) had high-energy fractures (e.g. falling from more than 1-m height, bicycle or motor vehicle accident).

The primary closed reduction was mainly carried out by orthopaedic and hand surgery residents. Immobilization was in a short arm cast, and radiological controls took place 1 and 2 weeks after repositioning. If the alignment was acceptable (< 2 mm radial shortening, < 10° dorsal tilt, < 1 mm step-off), cast immobilization was continued for 5–6 weeks. Patients with > 2 mm radial shortening, > 10° dorsal tilt, > 1 mm step-off seen in posteroanterior and lateral radiographs of the wrist were referred to surgical treatment.

**Fig. 1:**
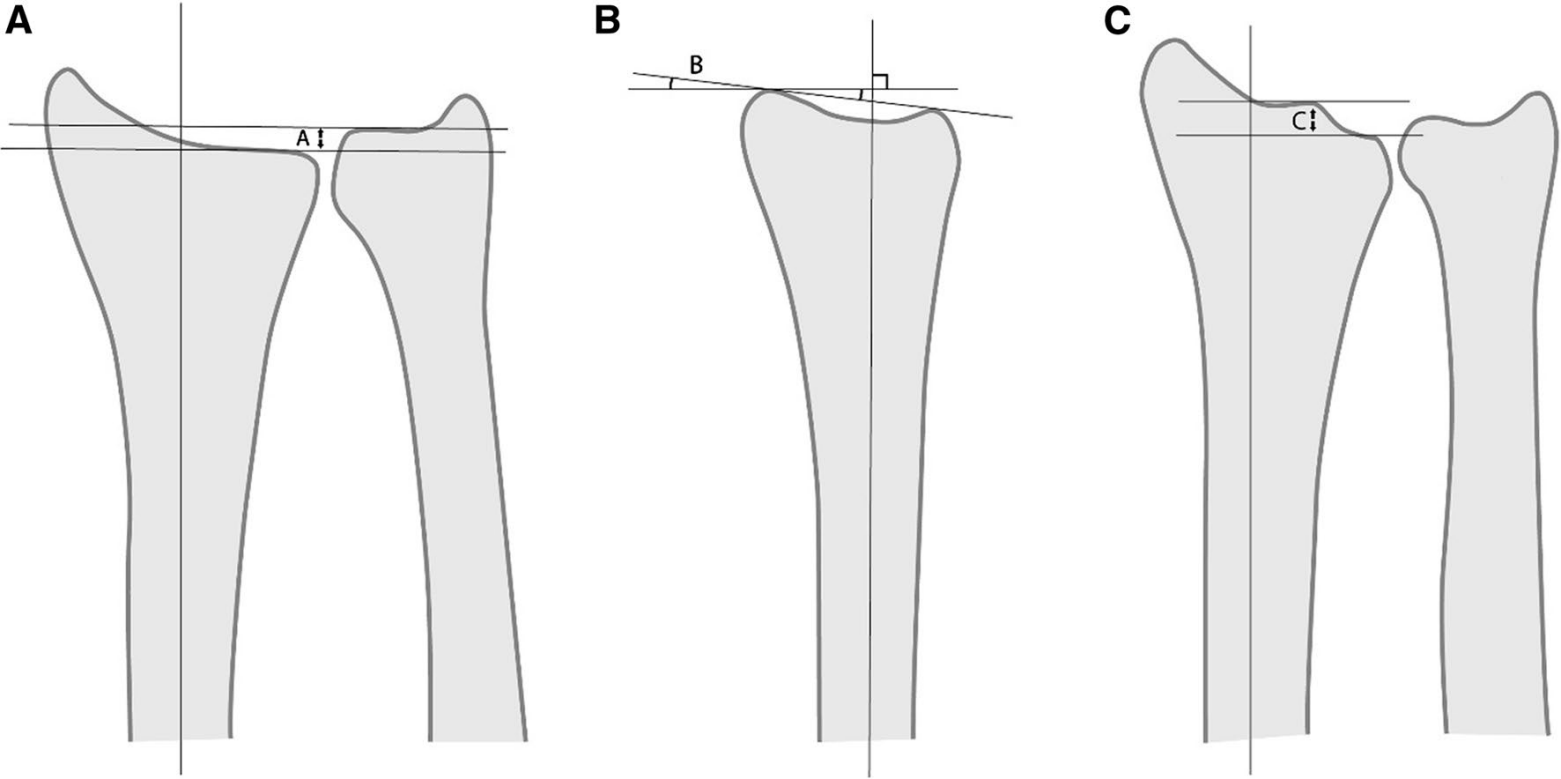
The following radiographic parameters were measured: **a** radius shortening compared with the ulna (**a** = mm). **b** Dorsal angulation (dorsal tilt) of the radius (**b** = °). **c** Step-off at joint surface (**c** = mm)

**Fig. 2:**
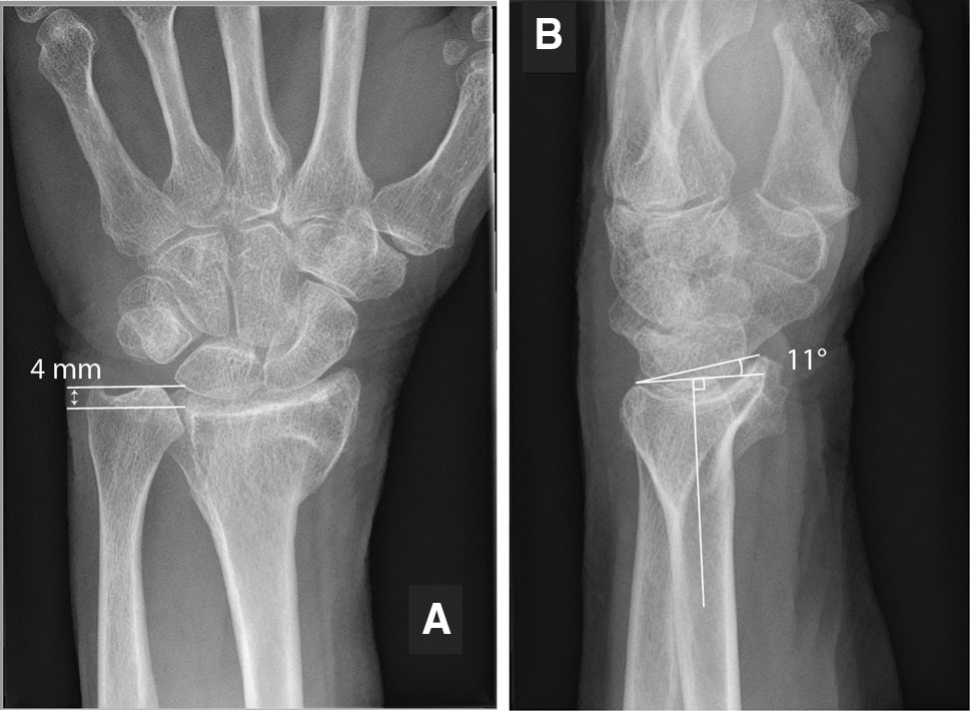
Mid-term radiological outcome of a 68-year-old woman. **a** Antero-posterior radiograph showing 4 mm of radial shortening, **b** lateral view showing 11° of dorsal tilt

Follow-up included standardized posteroanterior and lateral radiographs of the wrist. In the radiographs dorsal/volar abnormal angulation of the radius, radial shortening compared with the ulna, and possible incongruence (step-off) at joint level were measured (Figs. 1, 2). Radiological results were considered as well-aligned if they were ‘good’ (< 2 mm radial shortening, < 10° dorsal tilt, < 1 mm step-off) or ‘exact’ (no shortening, tilt or step-off).

Prior to a clinical examination, the patients completed the Finnish version of the Quick Disabilities of Arm, Shoulder and Hand (QDash) questionnaire, and the Patient-Rated Wrist Evaluation (PRWE) questionnaire. QDash is an 11-item questionnaire in which the upper extremity is considered to be a single functional unit. The PRWE questionnaire has 15 items designed to measure wrist pain and disability in activities of daily living. Both are scored from 0 (best) to 100 (worst).

The clinical examination and radiographic evaluation were performed by an experienced consultant hand surgeon (LR). Measurements of wrist range of motion (ROM) were taken with a hand-held goniometer: active extension (0°–70°), flexion (0°–70°), supination (0°–90°) and pronation (0°–90°). Grip strength (kg) was determined using a dynamometer (JAMAR hand dynamometer model J00105, Lafayette, IN 47903, USA). Both ROM and grip strength were compared with those on the contralateral unaffected side. Pain was measured on a visual analogue scale (VAS; 0–10) and on PRWE pain subscale. All complications and later operations were recorded.

We investigated patient-rated outcome measures (PROms), PRWE, QDash and VAS scores, plus clinical evaluation with ROM measures, and radiological outcome. The Finnish versions of the PRWE and QDash questionnaires have been culturally adapted and validated [18, 19].

Clinical outcomes in cases of shortening and dorsal/volar tilt were analysed separately. Concerning shortening of the radius, the cases were divided into two subgroups, those with shortening of 2 mm or more (34 cases) and those with less than 2 mm shortening or no shortening at all (34 cases).

The data were entered and analysed using the Statistical Package for the Social Sciences (IBM SPSS Statistics 25.0, IBM, Somers, IL, USA). The results are presented as means and standard deviations, medians and ranges, or the number of patients, as appropriate. The Mann–Whitney *U*-test was used for non-normally distributed continuous and ordinal data. Correlations between radiological malunion (dorsal tilt, articular step-off and radial shortening) and ROM values (flexion–extension, pronation–supination) were calculated using Spearman’s rank correlation. A two-sided *p* value of less than 0.05 was considered statistically significant.

## Results

The demographic characteristics of the study patients are presented in Table 1. All patients were treated by means of closed reduction and below-the-elbow cast immobilization for 5 weeks (mean 5.1, SD 0.7). The demographic characteristics did not statistically differ between participants and patients that declined or could not participate into the follow-up study.

**Table 1:** Demographic data of patients with conservatively treated C-type^a^ distal radius fractures

	Female *n*	Male *n*	Total *n*
Number of patients (%)	43 (65%)	23 (35%)	66 (68 wrists)
Age at injury (mean, SD)	55.0 (13.4)	47.8 (15.5)	52.9 (14.1)
Age at follow-up (mean, SD)	61.4 (13.5)	55.0 (15.7)	59.3 (14.2)
Dominant hand injury (%)	23 (52%)	13 (54%)	36 (54%)

^a^According to AO classification Müller et al. [20]

At the time of the follow-up appointment, at a mean of 6.7 years (SD 0.5, range 5.8–7.7) after the primary treatment, deterioration of the achieved primary alignment was found in 46 of 68 wrists (68%). The patients had either ≥ 2 mm shortening of the radius (*n* = 34, 51%), ≥ 10° dorsal tilt (*n* = 22, 33%) or step-off at the joint surface (*n* = 4, 6%), or a combination of these three. Malalignment of any kind was found in 52 cases (76%), versus exact anatomical alignment (16 wrists, 24%). We found no statistically significant differences in QDash (mean 10.3, SD 8.2 vs. 5.7, SD 8.2, *p* = 0.213) or PRWE scores (mean 8.1, SD 10.9 vs. 5.1 SD 8.9, *p* = 0.126) between the patients with radiologically malaligned versus well-aligned (good or exact) wrists, respectively.

Group analysis concerned shortening, dorsal tilt and incongruity of the joint surface. In cases of shortening of the radius (mean 4.2 mm, SD 1.5), no statistically significant differences in PROms were seen between malaligned and well-aligned groups (QDash 12.8, SD 18.6 vs. 5.5, SD 6.3, *p* = 0.220; PRWE 9.1, SD 12.7 vs. 5.7, SD 7.7, *p* = 0.40). Neither did ≥ 10° dorsal tilt of the radius (mean 16°, SD 7.6) show an association with worse QDash scores (mean 14.7, SD 21.1 vs. 6.5, SD 8.8, *p* = 0.241) or PRWE (mean 10.1, SD 12.8 vs. 5.8, SD 9.2, *p* = 0.226) compared with patients with anatomical radiographic alignment (Table 2). Volar tilt of the radius was seen in five cases (mean 20°, SD 4.5°, range 15°–25°), with no statistically significant difference in PROms compared with patients with normal anatomy. Step-off at the joint surface was measured in ten cases (mean 1.4 mm, SD 0.5, range 1–2 mm) after the primary closed reduction, but in only four cases at follow-up.

**Table 2:** Association between radiological and functional outcome 6.7 years after conservative treatment

	QDash mean (SD)	*p* value	PRWE mean (SD)	*p* value
*Shortening of radius*				
Radius shortened ≥ 2 mm, *n* = 34	12.8 (18.6)	0.22	9.1 (12.7)	0.40
No shortening or < 2 mm, *n* = 34	5.5 (6.3)		5.7 (7.7)	
*Dorsal tilt of radius*				
Dorsal tilt ≥ 10°, *n* = 22	14.7 (21.1)	0.241	10.1 (12.8)	0.226
No dorsal tilt or < 10°, *n* = 46	6.5 (8.8)		5.8 (9.2)	

*QDash* Quick Disabilities of the Arm, Shoulder and Hand, *PRWE* Patient-Rated Wrist Evaluation

Restriction of ROM was found in 43% of the wrists (*n* = 29). There was a constraint in wrist extension in 27 cases (39%; mean 13°, SD 8.3, range 5°–45°) compared with the contralateral wrist. A flexion deficit was found in 29 (43%) wrists (mean 10°, SD 6.3°, range 5°–20°), a supination deficit in 11 (16%) wrists (mean 10°, SD 6.3°, range 10°–20°) and a pronation deficit in three wrists (mean 8°, SD 1.8°, range 5°–10°). While radiological alignment had no statistically significant effect on PROms, a deficit in ROM was significantly associated with increased QDash and PRWE scores (Table 3). Patients suffering from extension deficit versus full extension reported poorer QDash scores (mean 15.9, SD 20.2 vs. mean 5.0, SD 6.2, *p* = 0.037). In addition, patients with flexion deficit had worse PRWE scores versus patients with no deficit (mean 11.5, SD 13.6 vs. 4.4, SD 6.3, *p* = 0.005). Moreover, a deficit in supination was statistically significantly associated with impaired PROms compared with patients with a normal range of supination (QDash: mean 21.7, SD 25.2 vs. mean 6.8, SD 9.8, *p* = 0.025; PRWE: mean 18.9, SD 18.1 vs. 5.2, SD 6.6, *p* = 0.007). No statistically significant correlation was found between radiological malalignment (≥ 2 mm radius shortening, ≥ 10° dorsal tilt, ≥ 1 mm step-off) and a deficit in ROM.

**Table 3:** Associations between wrist range of movement and patient-rated outcome after distal radius fracture

	*N* (%)	Mean (SD)	QDash (SD)	*p* value	PRWE (SD)	*p* value
Extension deficit	27 (39%)	13° (8.3)	15.9 (20.2)		10.9 (14.0)	
No extension deficit	41 (61%)		5.0 (6.2)	0.037	5.1 (6.8)	0.18
Flexion deficit	29 (43%)	10° (6.3)	13.7 (18.7)		11.5 (13.6)	
No flexion deficit	39 (47%)		6.0 (9.3)	0.13	4.4 (6.3)	0.005
Supination deficit	11 (16%)	10° (6.3)	21.7 (25.2)		18.9 (18.1)	
No supination deficit	57 (84%)		6.8 (9.8)	0.025	5.2 (6.6)	0.007
Pronation deficit	3 (4%)	8° (1.8)	14.4 (12.9)		17.8 (13.2)	
No pronation deficit	65 (96%)		9.1 (14.6)		6.9 (10.3)	

*N* number of cases, *QDash* Quick Disabilities of the Arm, Shoulder and Hand, *PRWE* Patient-Rated Wrist Evaluation

Mean grip strength compared with the opposite side (bilateral fractures excluded) was 94% (SD 17%, 50–130%) and pain, measured by means of a VAS, was 0.9 (SD 1.2, 0–6.0). Neither grip strength nor pain scores (VAS or PRWE subscale) showed a significant difference when comparing well-aligned and malaligned wrists.

At the time of the follow-up appointment, 39 patients (of working age 39/41, 95%) were working normally, one 56-year-old man had appropriate arrangements in his work as a result of wrist problems and one 59-year-old female was unable to work, partly because of wrist problems, partly because of psoriarthritis. Twenty-four (36%) patients were already retired at the time of follow-up. The conservative treatment was not without any complications (*n* = 6, 9%): three patients developed carpal tunnel syndrome treated by means of surgical intervention later on, two patients suffered rupture of the extensor pollicis longus tendon, and one patient suffered from complex regional pain syndrome.

## Discussion

While it has become increasingly popular to treat distal radius fractures surgically, most radius fractures are treated conservatively with closed reduction and plaster immobilization [3]. Even though secondary displacement after closed reduction is common, there is a lack of evidence supporting guidance as regards surgical versus conservative treatments [10, 17, 20]. As radiographic indices may not predict functional outcome, it is imperative to assess the mid-term results of conservatively treated distal radius fractures, even in the context of unstable fracture patterns [7, 10, 16, 20].

As the complexity of fracture morphology increases, combined with deteriorating bone quality, it becomes more common to detect malunion of a primarily well-reduced fracture. Wadsten et al. [21] studied late displacement (after 10–14 days) during conservative treatment of type A2 to C3 AO-classified distal radius fractures. Fractures with good alignment without the need for primary reduction lost their position in 28% of cases. However, the risk of late displacement increased up to 52% in cases of initially displaced fractures. In a study by Jaremko et al. [16], 71% of 74 consecutive patients (> 50 years) with non-operatively managed distal radius fractures had at least one unacceptable radiographic deformity at cast removal 6 weeks post-reduction. Arora et al. [7] compared conservative versus ORIF treatment in patients older than 70 years. In this study, malunion occurred in 89% of primarily reduced fractures which were treated conservatively. In our present study of relatively unstable AO type-C distal radius fractures, 68% showed deterioration of the achieved primary alignment.

Elderly patients in particular have been reported to have satisfactory functional results despite imperfect radiological healing [7, 13, 14, 22]. In this paper, we studied patients between 18 and 73 years of age, with similar observations across the study cohort: shortening, or dorsal or volar tilt of the radius did not show a significant association with QDash or PRWE scores.

PROms scores were worse among patients with radiological malunion outside the accepted range of The Finnish national Current Care Guidelines (without statistical significance) compared with patients with radiological deformity within the acceptable range [11]. Jaremko et al. [16] reported a similar result in a cohort of 74 patients: none of the unacceptable radiological parameters showed a significant relationship with QDash scores. Moreover, Wadsten et al. [23] found no statistically significant association between QDash, EQ-5D or VAS scores and either minimally displaced or late-displaced distal radius fractures.

In our present study, restriction in range of motion was common though moderate in mid-term follow-up: 39% of the patients showed restriction in extension, and 43% in flexion. A pronation deficit was uncommon, while a supination deficit was seen in 16% of the patients. Restriction in ROM was significantly associated with worse PROms. However, the scores did not reflect an even minimal clinically important difference [24]. Similarly, Wilcke et al. [25] reviewed 78 patients with closed reduction and plaster immobilization or external fixation and found that objective physical results were associated with a better patient-rated outcome, as measured by DASH scores.

Step-off at the joint level was fairly unusual. Moreover, we found remodelling of the joint surface over time: incongruity was not found in six of ten joint step-offs at follow-up. Shortening of the radius of ≥ 2 mm was seen in 34 (50%) wrists. However, no radiographs of the opposite side were taken in order to detect any possible natural ulna plus variance.

Our hospital is a tertiary referral clinic for operative treatment, and we do not routinely offer physiotherapy to patients after cast immobilization, as late follow-up and introduction of hand therapy is offered in outpatient clinics. Adherence to hand therapy in studies of short-term outcome after distal radius fracture has been shown to be beneficial [26]. Thus, a means to increase adherence to outpatient hand therapy might prove to play a significant role in mid-term functional results and PROms as well.

Complications were infrequent in our study (6/68 wrists, 9%). Complications such as carpal tunnel syndrome and rupture of the EPL tendon could be associated with the radius fracture itself rather than the chosen treatment modality. The complication rate associated with surgical treatment has been reported to be 15–27% [7–9, 27, 28, 29]. Only two patients in our group had an impairment (unrelated to complications) that substantially limited their ability to return to their previous professions.

We acknowledge the limitations of our study. It is a retrospective case series in design without sample size determination. No PROms were recorded at the time of treatment. Furthermore, we studied all type-C distal radius fractures, from less comminuted C1 fractures to more unstable C2 and C3 fractures.

The strength of the study is the outcome appraisal (both radiological and clinical) in mid-term follow-up of all patients. The clinical follow-up examination was performed by an experienced hand surgeon. The follow-up time was long, being nearly 7 years.

## Conclusions

To conclude, deterioration of the achieved primary alignment was common in AO classification type-C radius fractures (here, in 68% of the wrists). However, this deterioration lacked (clinically) a significant association with patient-rated outcome measures (Patient-Rated Wrist Evaluation and QDash scores) in the mid-term.
